# Prevalence of Skin Injuries in Beach Volleyball Athletes in Greece

**DOI:** 10.3390/jcm13072115

**Published:** 2024-04-05

**Authors:** Efstathios Rallis, Niki Tertipi, Eleni Sfyri, Vasiliki Kefala

**Affiliations:** Department of Biomedical Sciences, School of Health Sciences, University of West Attica, GR-12243 Athens, Greece; erallis@uniwa.gr (E.R.); elsfiri@uniwa.gr (E.S.); valiakef@uniwa.gr (V.K.)

**Keywords:** skin injuries, beach volleyball athletes, epidemiology

## Abstract

**Background:** Skin injuries often affect the sports community. Almost every type of athletic activity is associated with traumatic skin injuries, such as surface wounds, bruising, abrasions, subcutaneous hematomas, blunt trauma, nail injuries, friction burns, and blisters. **Methods:** The aim of this study was to assess the rates, location sites, and seasons of appearance of skin injuries in beach volleyball athletes in Greece. Seven hundred and eighty-five beach volleyball athletes participated in this study. The average age was 28.4 years. Skin injuries included superficial wounds (erosions, incisions, lacerations), deep wounds, hematomas, nail lacerations, friction burns, and friction blisters. The recorded variables encompassed gender, age, the time of year when athletes might be at higher risk of injuries, and the specific body regions affected. Additionally, data regarding training details such as years of practice, weekly training frequency, and daily training duration were also documented. **Results:** Incidence rates correlated to gender: (a) superficial wounds (*p* < 0.001), (b) years of training: hematomas (*p* < 0.001), and (c) average hours of daily training: superficial wounds (*p* < 0.001), deep wounds (*p* < 0.001), and friction blisters (*p* < 0.001). **Conclusions:** Although early detection, recognition, and treatment are essential, the prevention of skin injuries can also be linked to health and athletic performance.

## 1. Introduction

According to the World Health Organization, an injury is defined as “any damage to tissues, internal or external, regardless of the cause that provoked it”. Injury or trauma is the sum of tissue damage caused immediately at the moment of the accident by various forms of mechanical agents when they exceed the natural resistance of tissues and organs. Almost every type of sporting activity is associated with traumatic skin injuries, such as superficial injuries, hematomas, deep wounds, nail lacerations, friction burns, and friction blisters. An athlete’s skin is particularly exposed to a wide range of repetitive, physical, environmental, or stress-inducing factors that affect its protective function. Skin traumatic injuries can be located on the feet in the form of blisters, calluses, corns, and onychodystrophies [1]. They usually involve the upper layers of the epidermis and, in some cases, may include the basal layer as well. Areas of the skin with injuries are characterized by bleeding, bloody crusts, and epithelialization, usually without scar formation but commonly leaving post-inflammatory hyperpigmentation [2].

Skin injuries in athletes frequently originate from the training environment. Physical activity-related injuries can compromise the skin and mucous membrane barriers, facilitating the entry of infectious agents [3,4]. Additionally, sand harbors pathogenic microbes that pose risks to human health and can result in severe infections [5,6]. In superficial wounds, there is a partial or complete disruption of the skin’s continuity, affecting its layers. These wounds are benign and typically only require antiseptic treatment and temporary dressing [7,8]. 

Beach volleyball enjoys significant popularity among young adults, but it also comes with a notable frequency of injuries [9]. Similar to other sports, beach volleyball poses a substantial risk of skin injuries. These injuries may result from cuts, scrapes, or abrasions, exposure to infectious agents and environmental elements, as well as physical contact between players [10]. Cuts, scrapes, and abrasions are frequent occurrences in beach volleyball due to the abrasive nature of sand and the quick movements required during play. Diving for the ball, sliding, and even just falling onto the sand can lead to skin abrasions and lacerations. These injuries can range from minor scratches to deeper wounds requiring medical attention.

The existing literature lacks comprehensive epidemiological data on traumatic skin injuries in volleyball athletes. There has been no prior descriptive epidemiological research referring to traumatic skin injuries in beach volleyball athletes. In this study, we evaluated skin injuries in beach volleyball athletes, which included superficial wounds (such as erosions, incisions, and lacerations), deep wounds, hematomas, nail lacerations, friction burns, and friction blisters. Preventing these injuries among athletes is essential to avoid unnecessary health issues and to minimize disruptions to their training and competition schedules.

## 2. Materials and Methods

In this study, we developed and distributed an online questionnaire via Google Forms. Before launching the survey, a pilot study was carried out to validate the accuracy and clarity of the questions. The test–retest method was used with the same sample of volleyball athletes. Approval for this research was granted by the Research Ethics Committee of the University of West Attica (20.07.2020/app. No. 48944) and the Hellenic Volleyball Federation (06.03.2019/app. No. 746).

The questionnaire’s content and data collection methods were carefully crafted to respect the privacy and dignity of all participants. Before participation, informed consent or assent was obtained from each individual. The Hellenic Volleyball Federation sent out the link to the Google Forms questionnaire in November 2020 directly to athletes. The questionnaire covered demographic information, such as gender and age, and inquiries concerning (a) traumatic skin injuries, specifically superficial injuries, deep wounds, hematomas, nail lacerations, friction burns, and friction blisters; (b) the timing of injury occurrence throughout the season; (c) the specific body areas affected by the injuries; and (d) practice specifics, covering the frequency of yearly, weekly, and daily training sessions, about the types of skin injuries observed.

### Statistical and Data Analysis

All the information gathered via the questionnaire was inputted into an electronic database constructed using Excel v16.0. Subsequent data analysis was executed utilizing IBM SPSS Statistics for Windows, version 26.0. Categorical variables were depicted as absolute (*n*) and relative (%) frequencies, whereas continuous variables were represented as means (standard deviation, SD) and/or medians (interquartile range, IR). The normality assumption was evaluated through the Kolmogorov–Smirnov criterion (*p* > 0.05 for all variables), histograms, and normal probability plots. Bivariate analyses were undertaken, employing the X^2^ test and the X^2^ trend test to ascertain associations among categorical variables, along with the Mann–Whitney test to probe differences between groups within continuous variables. A two-sided *p*-value of 0.05 was considered statistically significant. 

## 3. Results

### 3.1. Demographic Characteristics and Training Elements

The study population comprised 785 beach volleyball athletes. No invalid questionnaires were reported. Demographic characteristics and data on the participants’ training are presented in Table 1.

In total, 329 athletes were males, and the mean age of all study participants was 28.4 years (SD = 9.0). Among them, 384 (48.9%) athletes had less than 6 years of training in beach volleyball, while 401 (51.1%) had more than 6 years. A total of 697 (88.8%) athletes trained less than four times per week, and 11.2% trained more than four times a week. Finally, 65.1% trained for 1 to 2 h daily, 33.4% trained for more than 2 h, and 1.5% trained for less than 1 h.

### 3.2. Dermatological Diseases/Traumatic Skin Injuries, Season of Appearance, and Body Location

Overall, 502 athletes (63.9%) in the study population reported superficial injuries, 267 athletes (34%) deep wounds, 288 athletes (36.7%) hematomas, 211 athletes (26.9%) nail lacerations, 392 athletes (49.9%) friction burns, and 171 athletes (21.8%) friction blisters. Regarding body localization sites, for superficial injuries, the most commonly identified site was the lower limbs (75.3%). Athletes with deep wounds most frequently reported the lower limbs as the affected body location (59.3%). Among athletes who reported hematomas, the most commonly identified location was the lower limbs (78.1%). Those with nail lacerations commonly reported the lower limbs as the affected area (87.1%). Athletes with friction burns most commonly reported the upper limbs as the affected body location (58.9%), while those with friction blisters reported soles (52.6%) (see Table 2 and Table 3). 

### 3.3. Correlations

Bivariate analyses were carried out (as presented in Table 4, Table 5 and Table 6), with superficial injuries, deep wounds, hematomas, nail lacerations, friction burns, and friction blisters examined as the dependent variables. The independent variables included demographic characteristics, training experience duration, frequency of weekly training sessions, and average daily training duration.

Athletes who exhibited superficial injuries had a significantly higher percentage of females (*p* < 0.001), fewer than 6 years of training in beach volleyball (*p* = 0.004), and more than four weekly training sessions (*p* = 0.004) compared to athletes who did not experience such traumas. Athletes who trained less than four times a week showed a higher percentage of surficial injuries, 70.2%, compared to those who trained more than four times, 59.8%; OR = 1.17. It was 1.17 times more likely for those who trained less than four times a week to exhibit surface injuries (*p* < 0.05) compared to those who trained more than four times per week. 

The majority of athletes who reported deep wounds were females, had more than 6 years of occupation in beach volleyball, and underwent training more than four times per week compared to athletes who did not exhibit deep wounds. Athletes who trained for more than 2 h were 1.15 times more likely compared to those who trained for 1–2 h and 1.33 times more likely compared to those who trained for less than 1 h to exhibit a deep injury (*p* < 0.05). 

Athletes with hematomas were disproportionately female (*p* < 0.001), had less than 6 years of training, and trained more than four weekly training sessions than athletes without hematomas. 

Athletes who experienced nail lacerations were mostly males, had more than 6 years of training, and trained more than four times per week. In both traumatic injuries, the daily training time mostly was 1–2 h. 

The majority of athletes who referred friction burns were males, had less than 6 years of training, trained less than four times per week, and had an average daily training duration of 1–2 h compared to athletes with no friction burns. Males displayed a higher percentage of friction blisters, had less than 6 years of training, and trained more than four times per week with an average daily training duration of 1–2 h compared to athletes who did not have any friction blisters.

## 4. Discussion

As the athlete population has grown, there has been a corresponding rise in the incidence of skin injuries [9]. This study represents an inaugural study with epidemiological data about skin injuries among beach volleyball athletes. The previous literature has been limited to descriptive studies mainly focusing on musculoskeletal injuries [3,4,5,10,11,12]. In a recent study by us, viral skin infections were reported in beach volleyball athletes [13].

The main outcomes of this research encompassed the incidence rates of skin injuries and their precise anatomical sites. We explored the probability of skin injuries in relation to various factors such as age, gender, time of occurrence, injury site, years of experience, weekly training program, and average daily training duration. These data provided an assessment of skin injuries from beach volleyball athletes, which can contribute to understanding the prevalence of such traumas. We examined six different types of skin injuries: superficial injuries, deep wounds, hematomas, nail lacerations, friction burns, and friction blisters. According to our findings, the most common skin trauma was a superficial injury. 

### 4.1. Superficial Injuries

These lesions typically involve the upper layers of the skin and may, in some cases, include the basal layer as well. Areas of the skin with superficial injuries are characterized by erosions (abrasions), bleeding, and bloody blisters [2]. Accidental abrasions usually appear after falls and sports-related mishaps in children, whereas among older individuals, falls stand out as a prominent cause of such injuries [14].

Wounds with skin tearing are injuries caused by friction on the most superficial layers of the skin. In beach volleyball, this can occur from player collisions or contact with objects or surfaces, resulting in damage to the skin’s capillaries and the formation of an open wound. Proper care of open wounds can reduce healing time and prevent infection. The most commonly reported areas affected in athletes are the head, trunk, and lower extremities [15]. In beach volleyball athletes, injuries may occur regardless of gender and training duration. However, the frequency of injuries seems to be determined by gender, as male athletes are 1.5 times more likely to sustain injuries compared to female athletes [16].

In our study, males compared to females exhibited a higher incidence of superficial injuries, 68.1% versus 61%, and were 1.11 times more likely to develop superficial injuries, which agrees with Reitmayer [16]. 

During winter, we observed a higher incidence rate. The beach environment and the season are factors that may further exacerbate skin injuries. Exposure to cold can cause dryness, making the skin more vulnerable. These injuries were commonly visible on the upper and lower limbs, palms/hands, and corpus. Athletes who trained less than four times a week exhibited a higher rate of superficial injuries. This finding is likely attributed to the experience developed in athletes who train more to avoid movements that may lead to injury.

### 4.2. Hematomas/Nail Lacerations

Blood accumulation resulting from trauma may cause a hematoma. It can be subcutaneous, intramuscular, or occur within internal cavities, such as the abdomen, chest, or beneath the nail. Muscle hematomas usually occur after contusion or rupture [17]. A subungual hematoma is caused by trauma, is quite painful, and is due to violent compression or crushing of the nail bed of the finger or toe. 

In our study, the frequency of hematomas was higher in females, 36.8, was mostly seen on the upper limbs, lower limbs, and palms/hands, and was more common during summer and winter seasons. Muscle injuries, such as hematomas, are prevalent among athletes across different disciplines, with an occurrence rate reaching as high as 39.2% [17]. In the present study, it was observed that athletes who trained for less than six years had a higher incidence of hematomas, at 31.7%. 

Onycholysis, resulting from injury, is notably painful and occurs due to a forceful impact, such as a crushing injury to the nail phalanx of a finger or toe [1]. In our study, the prevalence of onycholysis among volleyball athletes was 24.6% in males and 30.1% in females and was not affected by the duration of training. However, it was reported that athletes who trained more than four times per week had a higher incidence of nail trauma, at 30.1%.

### 4.3. Deep Wounds

A deep wound is defined as a complete disruption of the continuity of the skin due to mechanical force. Minor, superficial wounds are also called abrasions or erosions, while serious, open wounds are referred to as traumatic wounds. Deep wound injuries to the skin and subsequent bleeding can occur in many sports including volleyball [15].

Physical activity-associated injuries can disrupt the skin’s natural protective barrier [18]. The sand covering a beach volleyball court can have different properties that may affect athletes’ movements during training and competitions. The requirements for the sand material according to the standards of the International Volleyball Federation (FIVB) concern the size of the grains to create suitable conditions for proper court drainage and avoid compression in case of rainfall. The sand must be free from debris or other harmful substances as potential causes of skin injuries. The grain size of the sand should not be too fine to avoid sticking to athletes’ skin, and it should not contain clay and mud. In international competitions, the quality of the sand is approved by the official FIVB laboratory [19]. 

In our study, females exhibited a higher rate of severe injuries, 34.2%, and years of training did not affect athletes’ injury rates. Athletes who trained more frequently per week reported more injuries. Deep or minor wounds may occur in beach volleyball from objects moving at high speed, such as the ball, contact of the athletes with each other, or athletes falling or slipping.

### 4.4. Friction Burns

These lesions typically occur in athletes engaged in sports as a result of multiple frictional forces acting on a repeatedly traumatized area of the skin. Friction or mechanical stress can lead to painful friction blisters, especially when there is excessive moisture or when the skin has not yet toughened to withstand this stress [18]. Friction burns are due to injuries, are common in a variety of indoor and outdoor sports and activities, and have been associated with baseball/softball, basketball, volleyball, gymnastics, and soccer. Sliding against a rough surface like grass, artificial grass, trampolines, gym floors, and sand can readily cause friction burns [20,21]. 

In our study, the rate of friction burns was high, and males were more susceptible to these lesions, 51.4%, irrespective of their years of training and the number of weekly training sessions. The high rate of friction burns was attributed to the minimal clothing that beach volleyball athletes wear. Hall et al. [22] reported that friction burns frequently occur in competitive athletes due to the application of multiple frictional forces on a specific area of the skin and usually happen during midday hours, probably due to the higher surface temperature of the sand.

### 4.5. Friction Blisters

Skin temperature can influence blister formation. A 4 °C increase due to metabolic activity that has been reported, the hyperemic response, and the frictional force developed on the skin increase sweat production and accelerate blister formation by 50% [22]. Blisters were the most common finding among marathon runners, with an incidence ranging from 0.2% to 39% in studies conducted from 1973 to 1994. The areas most commonly affected were the fingertips, soft areas of the sole, and the back of the heel [1]. In sports like baseball, softball, weightlifting, pole vaulting, rowing, shot put, and discus throw, athletes who require implements may develop blisters due to the friction generated between the sports implements and the hand [23,24,25]. Athletes are susceptible to friction blisters due to the friction generated between the sports implements and the hand. These sports include baseball, softball, weightlifting, pole vaulting, rowing, shot put, and discus [21,23,24]. Athletes susceptible to friction blisters are typically involved in sports involving frequent starting, stopping, and rapid changes in direction. These movements cause the feet to shift within the shoe, leading to friction between the sock and the foot. Such sports include basketball, football, soccer, volleyball, and tennis [23]. Such injuries in athletes need to be treated to prevent a decrease in competitiveness and potential absence from training sessions [25]. In beach volleyball, athletes are especially prone to developing hand blisters due to the friction between their hands and the ball, as well as the sand. 

In our study, males displayed a higher percentage of friction blisters, 23.4%, with an average daily training duration of 1–2 h compared to athletes who did not have any friction blisters. The area of the body where the highest number of friction blisters was the lower limbs, accounting for 44.9%.

Preventive measures to avoid skin injuries include the appropriate selection of clothing and equipment for athletic activities that may expose the skin to injury. According to FIVB’s (Fédération Internationale de Volleyball) Official Beach Volleyball Rules 2021–2024 [26], the following rules apply:

- The playing surface must be at least 40 cm deep, composed of fine loosely compacted grains, as flat and uniform as possible, and free of rocks, shells, and anything else that can represent risks of cuts or injuries to the players;
- The weather must not present any danger of injury to the players;
- A player’s equipment consists of shorts or a bathing suit. A jersey or “tank-top” is optional, except when specified in the Tournament Regulations. Players may wear a hat/head covering and must play barefoot.

Based on the results of our study and the Official Beach Volleyball Rules 2021–2024, it seems that additional protective measures, such as, for example, elbow or knee pads or elastic shocks, are not mandatory for beach volleyball athletes. 

Employing proper execution techniques during activities can also reduce the risk of skin injury in beach volleyball athletes, as well as avoiding equipment during activities that may cause skin injuries. Paying particular attention to environmental conditions such as temperature, humidity, and potential hazards (ice, hail) in the area is necessary to address the potential risks of skin injury.

Limitations of our study included the lack of information regarding the training conditions of the athletes and the fields that may potentially contribute to the reduction in skin injuries, such as sand quality and sand preservation. Moreover, our dataset lacked information regarding the duration of training missed due to injuries, making comparisons with findings from similar studies challenging. Skin injuries are typically not documented by other researchers, further complicating comparative analysis.

The current study was conducted through anonymous questionnaires during the COVID-19 period, in which restrictive measures were taken, banning all sports and limiting any possible personal communication. Data regarding the effectiveness of protective equipment and appropriate clothing will be addressed in a future study along with information from athletes about skincare practices, psychological well-being, and the relationship between sand quality and the frequency of injuries. A future, longitudinal study that follows athletes over time and incorporates qualitative data from athletes about their experiences with skin injuries would probably be of interest.

Undocumented skin injuries and their inadequate treatment can result in delayed wound healing, bacterial growth, and infection, negatively impacting the athlete’s general well-being and athletic performance [27].

## 5. Conclusions

Our findings suggest that persisting skin injuries afflict beach volleyball athletes. Any athlete with such injuries should be removed from competition and training until the affected area is safely covered with waterproof, self-adhesive dressings to prevent bodily fluid leakage (blood, serum). Sufficient understanding and promptly starting treatment aid are crucial in maintaining uninterrupted team workouts and competitions. 

## Figures and Tables

**Table 1:** Demographic characteristics and data regarding athletes’ training.

	*n*	%
Gender		
Male	329	41.9
Female	456	58.1
Age	28.4 ^a^	9.0 ^b^
Number of training years		
Less than 6	384	48.9
More than 6	401	51.1
Number of weekly trainings		
Less than 4	697	88.8
More than 4	88	11.2
Average hours of daily training		
Less than 1	12	1.5
1–2	511	65.1
More than 2	262	33.4

Values are presented as *n* (absolute) and % (relative) frequencies unless stated otherwise. ^a^ Mean value; ^b^ standard deviation (SD).

**Table 2:** Traumatic skin injuries.

		Traumatic Skin Injuries	
	Superficial Injuries *n* (%)	Deep Wounds *n* (%)	Hematomas *n* (%)
No	283 (36.1)	518 (66)	497 (63.3)
Yes	502 (63.9)	267 (34)	288 (36.7)
Season of injury appearance			
Winter	218 (58.9)	66 (49.3)	112 (63.9)
Spring	3 (30.5)	38 (28.4)	85 (48)
Summer	237 (64.1)	84 (62.7)	89 (50.3)
Autumn	113 (30.5)	40 (29.9)	70 (39.5)
Body location			
Upper limbs	225 (50.6)	40 (24.71)	102 (42.1)
Lower limbs	335 (75.3)	96 (59.3)	189 (78.1)
Head	10 (2.2)	2 (1.2)	3 (1.2)
Corpus	105 (23.6)	44 (27.2)	44 (18.1)
Palms/Hand	85 (19.1)	8 (4.9)	20 (8.3)
Soles	121 (27.2)	43 (36.5)	28 (11.6)
Face	25 (5.6)	4 (2.5)	2 (0.8)

Values are expressed as *n* (absolute) and % (relative) frequencies.

**Table 3:** Traumatic skin injuries.

		Traumatic Skin Injuries	
	Nail Lacerations *n* (%)	Friction Burns *n* (%)	Friction Blisters *n* (%)
No	574 (73.1)	393 (50.1)	614 (78.2)
Yes	211 (26.9)	392 (49.9)	171 (21.8)
Season of injury appearance			
Winter	34 (47.2)	127 (52)	17 (28.3)
Spring	29 (40.3)	91 (37.3)	24 (40)
Summer	43 (59.7)	167 (68.4)	47 (78.3)
Autumn	30 (41.7)	126 (51.6)	20 (33.3)
Body location			
Upper limbs	25 (29.4)	169 (58.9)	15 (19.2)
Lower limbs	74 (87.1)	151 (52.9)	35 (44.9)
Head	0	2 (0.7)	0
Corpus	0	109 (38)	9 (11.5)
Palms/Hand	0	48 (16.7)	16 (20.5)
Soles	0	65 (22.6)	41 (52.6)
Face	0	13 (4.5)	0

Values are expressed as *n* (absolute) and % (relative) frequencies.

**Table 4:** Bivariate analyses using superficial injuries and deep wounds as the dependent variables.

	Superficial Injuries			Deep Wounds		
Characteristic	No	Yes	*p*-Value	No	Yes	*p*-Value
Gender			<0.001 ^a^			<0.001 ^a^
Male	105 (31.9)	224 (68.1)		218 (66.3)	111 (33.7)	
Female	178 (39)	278 (61)		300 (65.8)	156 (34.2)	
Age ^b^	25.0 (16)	30.0 (10)	0.001 ^c^	25.0 (16)	30.5 (10)	<0.001 ^c^
Number of training years			0.004 ^d^			<0.004 ^d^
Less than 6	219 (35.3)	402 (64.7)		401 (64.6)	202 (35.4)	
More than 6	33 (36.3)	58 (63.7)		62 (68.1)	29 (31.9)	
Number of weekly trainings			0.004 ^d^			0.010 ^d^
Less than 4	97 (29.8)	229 (70.2)		218 (66.9)	108 (33.1)	
More than 4	155 (40.2)	231 (59.8)		245 (63.5)	141 (36.5)	
Average hours of daily training			0.001 ^d^			0.001 ^d^
Less than 1	5 (41.7)	7 (58.3)		8 (66.7)	4 (33.3)	
1–2	161 (34.8)	301 (65.2)		285 (61.7)	177 (38.3)	
More than 2	86 (36.1)	152 (63.9)		170 (71.4)	68 (28.6)	

Values are expressed as *n* (%) unless stated otherwise. ^a^ X^2^ test. ^b^ Median value (IR). ^c^ Mann–Whitney test. ^d^ X^2^ test for trend.

**Table 5:** Bivariate analyses using deep wounds and nail lacerations as the dependent variables.

	Hematomas			Nail Lacerations		
Characteristic	No	Yes	*p*-Value	No	Yes	*p*-Value
Gender			<0.001 ^a^			<0.001 ^a^
Male	209 (63.5)	120 (36.5)		230 (69.9)	99 (30.1)	
Female	288 (63.2)	168 (36.8)		344 (75.4)	112 (24.6)	
Age ^b^	25.0 (16)	30.5 (9)	0.001 ^c^	25.0 (16)	30.5 (9)	<0.001 ^c^
Number of training years			0.001 ^d^			<0.001 ^d^
Less than 6	424 (68.3)	197 (31.7)		446 (71.8)	175 (28.2)	
More than 6	72 (79.1)	19 (20.9)		70 (76.9)	21 (23.1)	
Number of weekly trainings			0.100 ^d^			0.101 ^d^
Less than 4	213 (65.3)	113 (34.7)		246 (75.5)	80 (24.5)	
More than 4	229 (59.3)	157 (40.7)		270 (69.9)	116 (30.1)	
Average hours of daily training			0.006 ^d^			0.204 ^d^
Less than 1	9 (75)	3 (25)		9 (75)	3 (25)	
1–2	311 (67.3)	151 (32.7)		325 (70.3)	137 (29.7)	
More than 2	176 (73.9)	62 (26.1)		182 (76.5)	56 (23.5)	

Values are expressed as *n* (%) unless stated otherwise. ^a^ X^2^ test. ^b^ Median value (IR). ^c^ Mann–Whitney test. ^d^ X^2^ test for trend.

**Table 6:** Bivariate analyses using friction burns and friction blisters as the dependent variables.

	Friction Burns			Friction Blisters		
Characteristic	No	Yes	*p*-Value	No	Yes	*p*-Value
Gender			<0.001 ^a^			<0.001 ^a^
Male	160 (48.6)	169 (51.4)		252 (76.6)	77 (23.4)	
Female	233 (51.1)	233 (48.9)		362 (79.4)	94 (20.6)	
Age ^b^	25.0 (16)	30.0 (10)	0.001 ^c^	25.0 (16)	30.5 (9)	<0.001 ^c^
Number of training years			0.004 ^d^			<0.001 ^d^
Less than 6	289 (46.5)	332 (53.5)		470 (75.7)	151 (24.3)	
More than 6	50 (54.9)	41 (45.1)		75 (82.4)	16 (17.6)	
Number of weekly trainings			0.010 ^d^			0.185 ^d^
Less than 4	151 (46.3)	175 (53.7)		257 (78.8)	69 (21.2)	
More than 4	188 (48.7)	198 (51.3)		288 (74.6)	98 (25.4)	
Average hours of daily training			0.319 ^d^			0.001 ^d^
Less than 1	8 (66.7)	4 (33.3)		9 (75)	3 (25)	
1–2	214 (46.3)	248 (53.7)		337 (72.9)	125 (27.1)	
More than 2	117 (49.2)	121 (50.8)		199 (83.6)	39 (16.4)	

Values are expressed as *n* (%) unless stated otherwise. ^a^ X^2^ test. ^b^ Median value (IR). ^c^ Mann–Whitney test. ^d^ X^2^ test for trend.

## Data Availability

The data presented in this study are available on request from the corresponding author. The data are not publicly available due to privacy.
